# Stress Memory of Recurrent Environmental Challenges in Marine Invasive Species: *Ciona robusta* as a Case Study

**DOI:** 10.3389/fphys.2020.00094

**Published:** 2020-02-13

**Authors:** Hanxi Li, Xuena Huang, Aibin Zhan

**Affiliations:** ^1^Key Laboratory of Environmental Biotechnology, Research Center for Eco-Environmental Sciences, Chinese Academy of Sciences, Beijing, China; ^2^College of Resources and Environment, University of the Chinese Academy of Sciences, Chinese Academy of Sciences, Beijing, China

**Keywords:** physiology plasticity, environmental stress, ascidian, antioxidant defense system, stress memory, adaptive homeostasis

## Abstract

Fluctuating environmental changes impose tremendous stresses on sessile organisms in marine ecosystems, in turn, organisms develop complex response mechanisms to keep adaptive homeostasis for survival. Physiological plasticity is one of the primary lines of defense against environmental challenges, and such defense often relies on the antioxidant defense system (ADS). Hence, it is imperative to understand response mechanisms of ADS to fluctuating environments. Invasive species provide excellent models to study how species cope with environmental stresses, as invasive species encounter sudden, and often recurrent, extensive environmental challenges during the whole invasion process. Here, we studied the roles of ADS on rapid response to recurrent cold challenges in a highly invasive tunicate (*Ciona robusta*) by simulating cold stresses during its invasion process. We assessed antioxidative indicators, including malondialdehyde (MDA), total antioxidant capacity (T-AOC), superoxide dismutase (SOD), catalase (CAT), and glutathione (GSH), as well as transcriptional changes of ADS-related genes to reveal the physiological plasticity under recurring cold stresses. Our results demonstrated that physiological homeostasis relied on the resilience of ADS, which further accordingly tuned antioxidant activity and gene expression to changing environments. The initial cold stress remodeled baselines of ADS to promote the development of stress memory, and subsequent stress memory largely decreased the physiological response to recurrent environmental challenges. All results here suggest that *C. robusta* could develop stress memory to maintain physiological homeostasis in changing or harsh environments. The results obtained in this study provide new insights into the mechanism of rapid physiological adaption during biological invasions.

## Introduction

Nowadays, organisms often experience frequent and intensive environmental challenges derived from multiple interacting stressors, such as global climate change and habitat transitions influenced by increasing human activities (e.g., biological invasions; Pickering et al., 2013; Calosi et al., 2017). Amongst several strategies for mitigating those stresses, physiological plasticity is a crucial mechanism to tolerate fluctuating environmental changes (Somero, 2012; Jimenez et al., 2015; Spicer et al., 2019). It is incontrovertible that the adaptation within one generation is critically important to buffer environmental stresses to ensure survival, which is the prerequisite for subsequent adaptation and evolution of a population (Carlson et al., 2014). Rapid physiological regulation serves as one of the most direct and effective ways to mitigate threats to the survival of a species within one generation (Hofmann and Todgham, 2010; Seebacher et al., 2014). Thus, understanding physiological response dynamics is crucial for deeply investigating the homeostasis maintenance during environmental challenges and further predicting the influence of environmental changes on the survival and geographical distribution shifts of a species (Hoffmann and Sgro, 2011; Nandamuri et al., 2017).

Rapid environmental changes often trigger excessive loads of reactive oxygen species (ROS) by boosting the mitochondrial respiratory activity, reduce the fluidity of the mitochondrial membrane, and ruin the electron transport chain (i.e., causing oxidative stress; Murphy, 2009). Antioxidant defense system (ADS), as the first defense line and one of the most vital physiological mechanisms, can effectively eliminate the imbalance state of redox and protect organismal homeostasis against environmental stresses (Storey, 1996; Jimenez et al., 2015; Calabrese and Mattson, 2017). By activating antioxidants which can catalyze the decomposition of ROS into less toxic molecules within seconds to minutes, organisms keep the content of free-radicals and reactive oxidants at appropriate levels during acute environmental challenges (Sthijns et al., 2016; Oliveira et al., 2018). On the other hand, ADS can change the synthesis rate and content of downstream genes, including most of antioxidants, by altering the expression of transcriptional factors (TF) in ADS-related signaling pathways (Zhang, 2006; Kensler et al., 2007; Shadel and Horvath, 2015). The nuclear factor erythroid-2-related factor 2 (Nrf2)-keap1 signaling pathway is one of the most relevant and extensively studied pathways in resistance to oxidative stresses (Nguyen et al., 2003; Ristow, 2014; Sies et al., 2017). Nrf2 is suppressed by bounding to Kelch-like epichlorohydrin associating protein1 (Keap1) in a normal condition and lose the ability of translocation to the nucleus. While under stresses, Keap1 is repressed and the released Nrf2 is subsequently translocated forward to the nucleus where it accumulates substantially to activate the expression of its target genes (Ma, 2013). By modulating the expression of its target genes involved in antioxidants, such as superoxide dismutase (SOD), glutathione peroxidase (GPx), catalase (CAT), phase II detoxifying enzyme (e.g., glutathione S-transferase, GST; heme oxygenase-1, HO-1) and other antioxidative compounds, the Nrf2-keap1 signaling pathway can decline the content of free-radicals and/or reactive oxidants to a harmless level, thus protecting organisms against various environmental stresses for maintaining redox homeostasis ultimately (Schieber and Chandel, 2014; Davies, 2016; Sparks et al., 2019).

Recent studies have demonstrated that previously encountered abiotic stresses could affect organismal response against later stresses (i.e., stress memory; Crisp et al., 2016). Available evidence showed that organisms could respond more effectively to recurring stimulus through two distinct types of stress memory: type I shows the enhanced response with a faster and/or stronger pattern, and such a type could provide the benefits of potentiation to subsequent challenges. Type II has a feature of increased tolerance with steadier and less intense response, and such a type can provide the benefits of tolerance during recurrent stresses (Avramova, 2015). For example, after being subjected to several dehydration/rehydration cycles, *Arabidopsis thaliana* and *Zea mays* (maize) showed the transcriptional stress memory mainly by producing stronger reactions to improve the ability of moisture retention when compared to unstressed plants (Ding et al., 2013; Virlouvet and Fromm, 2015). The red seaweed *Bangia fuscopurpurea* could alter specific membrane fatty acid composition to own heat memory and increase heat tolerance to protect against future heat stresses (Kishimoto et al., 2019). However, in marine ecosystems, we have very limited knowledge on the mechanism of physiological response based on ADS under fluctuating environments (except *Mytilus californianus*, Jimenez et al., 2015; *Crassostrea gigas*, Moreira et al., 2016).

Biological invasions provide an excellent “natural experiment” to study how species cope with environmental stresses, as invasive species encounter sudden, and often repeated, extensive environmental challenges during the invasion process (Zhan et al., 2015; Ni et al., 2018). Particularly for human activity-mediated introductions, invasive species often encounter environmental changes much greater and/or faster than what species can experience under natural conditions such as seasonal fluctuations (Zhan et al., 2015; Chen et al., 2018a). Taking shipping-mediated invasions as an example, environmental conditions can change dramatically in ballast tanks, with temperature suddenly dropped from 20°C to the freezing point, and this type of great changes can be recurrent during the whole shipping voyage (Briski et al., 2013; Chen et al., 2018b). The response to the former stress may cause stress memory *via* achieving a renewed balance of homeostasis and tolerance to influence subsequent stress response. Thus, invasive species represent good candidates to study stress memory, in turn, the obtained results on stress memory can largely promote our understanding of invasion success, particularly on mechanisms of physiologic plasticity under environmental challenges.

Ascidians are excellent models to investigate how species respond to repeated harsh environments in coastal ecosystems (Zhan et al., 2015; Huang et al., 2017). Survival of repeated acute stresses during the transport and establishment stages is an indispensable ability and prerequisite for successful invasions to new habitats in one generation (Lenz et al., 2011). Many invasive species, especially for sessile organisms such as ascidians, have no chance to moderate stresses *via* behaviors such as escape from harsh environments, but largely rely on strong adaptive homeostasis through pre-adaptation or physiological plasticity to maintain homeostasis (Richards et al., 2006; Bruce et al., 2007). The highly invasive ascidian *Ciona robusta*, which is presumably a native to Northern Atlantic, has successfully invaded many coasts globally (Carver et al., 2006; Rius et al., 2014). It has become a popular model animal for evolutionary biology and invasion science (Delsuc et al., 2006; Zhan et al., 2015). *C. robusta* can tolerate a wide temperature range from −1°C to 35°C (Dybern, 1965). Amazingly, as a simultaneous hermaphrodite, *C. robusta* can produce eggs under low temperature as low as −1°C (Carver et al., 2003). Hence, the capacity of cold tolerance makes *C. robusta* a good model to explore mechanisms of physiological response to recurrent cold stresses.

Here we use this model species to study physiological plasticity represented by the regulation of Nrf2-depended ADS at the levels of both enzyme activity and transcriptional synthesis in response to repeated cold stresses. Our study used two rounds of cold stress-recovery to simulate repeated environmental changes during the invasion process. We aim to (1) explore the response profile of ADS of *C. robusta* under two rounds of cold stresses, and (2) verify the existence of stress memory based on ADS by comparing different responses on the variation of antioxidant indicators and ADS-related genes expression. The results obtained here are expected to promote our understanding of the maintenance of physiological homeostasis in fluctuating environments and provide new insights into the rapid physiological adaption process during biological invasions.

## Materials and Methods

### Sample Collection and Acclimation

Adult ascidians were collected from scallop cages in the Longwangtang maricultural area of Dalian, Liaoning Province, China (38°48′53″ N, 121°24′06″ E) in September 2018. Ascidians were transported into tanks in the laboratory and acclimated for one week at the temperature of 20 ± 1°C according to the field condition. In the laboratory, all animals were fed twice daily with algae powder mixture of wall-broken Chlorella (*Chlorella* spp.) and spirulina (*Arthrospira platensis*). At the end of the acclimation, 12 adults were randomly chosen as the unstressed group.

### Cold Stress Treatment and Recovery

**FIGURE 1:**
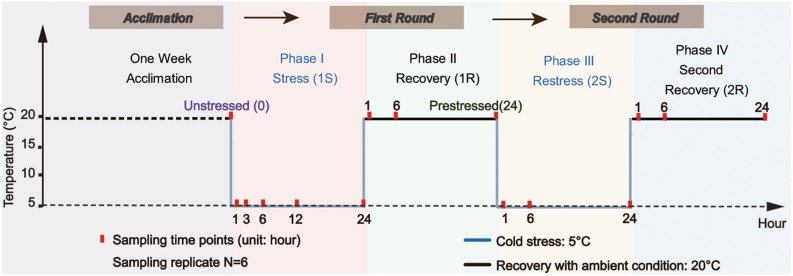
Experimental design scheme. Ascidians were acclimated for one week and then transferred to the temperature-controlled aquarium at 5 ± 1°C for cold stress treatment (1S). After 24-hour treatment, the temperature was recovered to 20 ± 1°C and lasted for another 24-hour as the first recovery stage (1R). The ascidians that experienced the first round of stress-recovery treatment were then subjected to the new round of stress-recovery experiment, which was exactly the same as the Round I. Red points indicate the time of sampling.

We used two cycles of “cold stress-recovery” experiment to simulate the recurring environmental stresses during the invasion process. The whole experiment consisted of two rounds (Round I and II) of challenges, each round included two phases of cold stress (1S and 2S) and recovery to ambient condition (1R, 2R), and every single phase lasted 24 h based on surveys from our former study (Huang et al., 2018). In order to clearly describe the comparison between response within two rounds, we defined samples from the ambient condition as “unstressed” and the final status of the first round (1R-24) as “prestressed.” The temperature of cold stress was set at 5 ± 1°C according to the minimum temperature within the normal tolerance range and the recovery temperature was 20 ± 1°C (i.e., field condition, see Figure 1 for the experimental design).

During the first stress (1S), 200 adult ascidians with similar body size were transferred from the acclimation tank at 20 ± 1°C to the temperature-controlled aquarium (80 cm × 45 cm × 40 cm) at 5 ± 1°C for the cold challenge. The density of ascidians was approximately 1.4 ind/L. After 24 h of cold exposure, the temperature was recovered to 20 ± 1°C and lasted for 24 h as the first recovery phrase (1R). The treated ascidians by the first round of stress-recovery were then subjected to a new round of stress-recovery experiment. Six individuals were randomly sampled at different sampling time points including 1, 6, and 24 h at each of the four phases (Figure 1). To obtain an accurate profile of ADS changes during acute cold stresses, we also sampled two additional points at 3-hour (1S-3) and 12-hour (1S-12) of the first phase (Figure 1). Ascidians were dissected on ice-chilled culture dishes and separated into two parts, including 100–150 mg pharynx muscle mass for RNA isolation and the rest of the pharynx muscle for physiological analyses for each individual. After dissection, all samples were flash-frozen in liquid nitrogen and stored at −80°C. Species identification of all samples was conducted according to Zhan et al. (2010) and Smith et al. (2012) to avoid species confusion due to the morphological similarity among *Ciona* ascidians.

### Antioxidant Status Assay

#### Malondialdehyde (MDA) Content and Total Antioxidant Capacity (T-AOC) Assay

Tissue supernatant was prepared for each bioindicator analysis. Each sample (80 mg pharynx muscle) was individually prepared in 0.9% sodium chloride solution with the proportion ratio of 1: 9 (w/v) and homogenized by tissue homogenizer in ice bath. Smear test was taken after homogenization to confirm the accomplishment of cell disruption. Subsequently, the homogenate was centrifuged at 10,000 *g* for 20 min under 4°C. Supernatants were immediately used for the determination of the total protein (TP), MDA concentration, T-AOC, special total SOD activity, special CAT activity and GSH concentration. All the following indices for specific enzymatic activity were defined as the concentration/activity unit of 1 mg tissue protein. TP concentration was determined using the Kaumas blue method-based kit (Nanjing Jiancheng, China).

The comprehensive and accurate assessment of antioxidant degrees was measured by both cellular damage products and antioxidant capacity. The cellular damage level was represented by the extent of lipid peroxidation, which was further indicated by the concentration of MDA using the thiobarbituric acid (TBA) method-based kit (Nanjing Jiancheng, China). T-AOC was measured by the assay kit (Nanjing Jiancheng, China) according to the instructions.

#### Antioxidant Enzyme Activity Assay

Animals rely on robust ADS to maintain the redox balance, including antioxidase system and other non-enzymatic antioxidant molecules. In this study, we selected three types of antioxidants, including total superoxide dismutase (T-SOD), catalase (CAT), and reduced glutathione (GSH). These indicators were tested by relevant assay kits (Nanjing Jiancheng, China). T-SOD was detected by the hydroxylamine method, whereas CAT and GSH were measured by the ultraviolet method and dithiol dinitrobenzoic acid method, respectively. All indicators mentioned above were measured by an ultraviolet spectrophotometer (DR6000, HACH Inc., United States).

### Total RNA Isolation and RT-PCR

Total RNA was extracted from the muscle tissue (50–100 mg) by TRIzol reagent (Ambion, United States) with the standard protocol. RNA integrity was tested by agarose gel electrophoresis and RNA concentration was determined by Nano 2000 spectrophotometer (Nanodrop Technologies, United States). A total of 3 μg high-quality RNA was reverse-transcribed to cDNA using the PrimerScriptII cDNA Synthesis Kit (Takara, Japan). Then cDNA was treated with RNase-free DNase I (Promega, United States) to avoid genomic DNA contamination, and 10-fold cDNA dilution was applied in downstream assays.

We assessed the gene expression of two transcription factors (Nrf2 and Keap1) and their target genes including Cu/ZnSOD, MnSOD, CAT, GPX, and GST to monitor the transcriptional change under repeated cold stresses. The β-actin gene was chosen as the reference gene for gene expression normalization (Fujikawa et al., 2010; Huang et al., 2016; Huang et al., 2019). The sequences of all genes were downloaded from Ensembl^1^ and all primers were designed by Primer Premier 5.0^2^. Primers were tested to ensure specificity and efficiency, and we used the primer pairs with the amplification efficiency of 1.9-2.1 for downstream analyses (Table 1).

**TABLE 1:** Primer sequences for the genes used in the real-time PCR.

Gene abbr.	Gene ID	Forward primer (5′–3′)	Reverse primer (5′–3′)	PCR efficiency
β-actin	ENSCING00000008797	ACCGGCTTCAGCTGCTAAGTAAC	TGTCCGATGTATTGGTCCTTTATTG	1.93
MnSOD	ENSCING00000004261	GTTGGGTCTGGATAAAGAAGCC	TTTGGACGGAAGTTCTTGTATTGT	2.00
Cu/ZnSOD	ENSCING00000021568	GACGGCAGATTCAAGTGGTGTAG	TGTTGTCTTGCTTTGTGGATGAG	1.98
CAT	ENSCING00000005187	TCGTGGGTTCGCCATCAAGT	TAAGATGAGTTTGCGGGTTTCG	1.92
GST	ENSCING00000003309	TCTTTATGTTTCGCTTGCTTGTC	GATCTTTCTCGCATTGCTCAAC	1.91
GPX	ENSCING00000012924	ATCGGAAAGGTTTCACTCGTTG	CTGATTACACGGGAAAGCAAGG	2.00
Nrf2	ENSCING00000003373	AACTCGGACACTGACGAAGCAT	TTTATGGAGAACGGAAGGACAAC	1.97
Keap1	ENSCING00000008356	CTTGGTCTTGCTGCGTTTGC	CTTCCGATTGCGACACCTGTT	1.94

Real-time PCR (RT-RCR) was conducted on Roche LightCycler^®^ 96 detection system (Roche Applied Science, Germany) by using FastStart Essential DNA Green Master (Roche Applied Science, Germany). A 20 μl reaction mix was composed of 10 μl SYBR Green Master Mix, 8 μl PCR-free water, 0.5 μl of forward and reverse primers and 1 μl cDNA template. PCR program consisted of 40 cycles with 95°C for 10 s, 60°C for 10 s and 72°C for 15 s. Negative controls with non-template were used to eliminate contamination of the reaction system during the whole experiment process. Relative gene expression of treated groups to corresponding unstressed groups was normalized by the 2^–ΔΔcq^ method (Pfaffl, 2001). For each gene, the transcriptional abundance of unstressed group was standardized as 1.0.

### Data Analysis

All data including antioxidative indicators and gene expression was described as mean ± standard error. The significance test of all pairwise comparisons between treated and unstressed groups was analyzed using One-way ANOVA after normal test and homogeneity of variance by Kolmogorov-Smirnov test in SPSS (Version 25.0, SPSS Inc., United States). After One-way ANOVA with stress time as the factor for each treatment group, the significance test between unstressed and treated groups was assessed by Least Significant Difference (LSD) *post hoc* test. Principal Component Analysis (PCA) was also conducted using the IBM SPSS Statistics 25 and all statistical graphs were generated by OriginPro 9.1.

The antioxidant response element (ARE), as the main DNA binding sequence of Nrf2, was predicted within 2,000 bp upstream of the transcriptional start site of each gene with MatInspector tool in Genomatix Software Suite^3^. We focused on potential Nrf2 target genes recognized by ARE core sequence (5′-G/ATGACNNNGC-3′), and then described the number, distribution, and correlation with ARE for each gene.

## Results

### Antioxidative Indicators

#### General Change Patterns of Antioxidative Indicators

**FIGURE 2:**
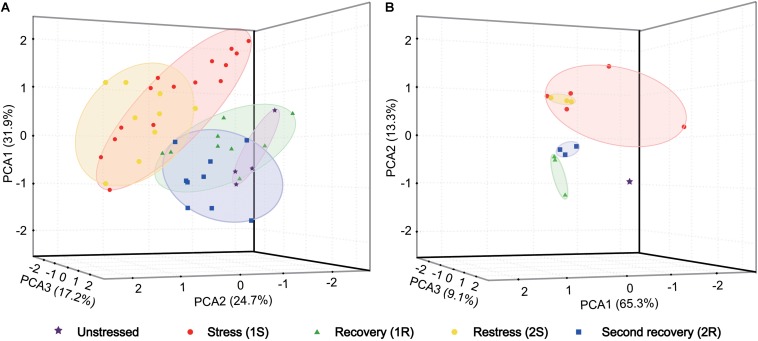
Principal component analysis (PCA) based on antioxidant indicators **(A)** and gene expression data **(B)**. Each data point represents a sample plotted using the first three principal components and the coordinate axis represents the first three principal component dimensions and their contribution to the overall variation among individuals. PCA was carried out on the log-transformed mean-centered data matrix.

We first explored changes of antioxidative indicators by principal component analysis (PCA) to identify the major variation among all samples. Three axes explained 73.8% of the total variation in organismal antioxidant capacity (Figure 2A). The PCA loading plot showed that the ascidians collected in Round I did not separate from those sampled in Round II. The samples collected in two recovery phases were separated from those under cold stresses, but were not separated from samples collected under unstressed condition. These results illustrated that cold challenge was the dominant source of changes on antioxidative indicators, and *C. robusta* should use different antioxidative defense strategies at the stress and recovery stages.

#### Response of Organismal Antioxidative Status

**FIGURE 3:**
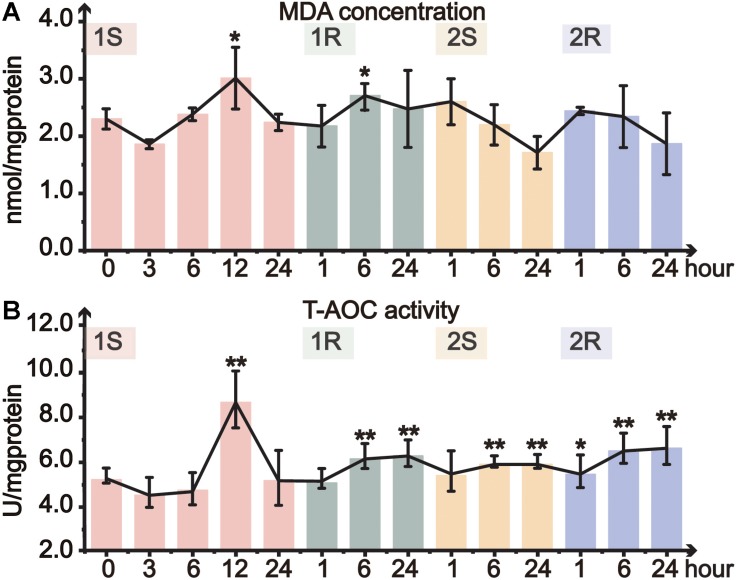
Organismal antioxidative level of *Ciona robusta* under unstressed (20°C), cold challenge (5°C) and recovery (20°C). **(A)** MDA (malondialdehyde) concentration; **(B)** T-AOC (total antioxidative capacity) activity. Each error bar represents the mean ± SE (*N* = 6). *p < 0.05, **p < 0.01.

Oxidative status of ascidians was assessed by MDA content and T-AOC. MDA content, which commonly indicates lipid peroxidation of the cell membrane, significantly increased at 1S-12 and 1R-6 and then returned to the unstressed level (Figure 3A). However, MDA content did not change in the subsequent round of cold stress. Our results showed a relatively stable status of MDA content without significant changes except for 1S-12, and such results suggest that *C. robusta* either be able to withstand the cold environment and/or have initiated ADS to maintain membrane integrity.

Total antioxidant capacity is a comprehensive indicator of antioxidative ability. During the first cold stress, T-AOC significantly increased at 12 h, and then returned to the control level at the end of the first stress (Figure 3B). In the following first recovery, T-AOC significantly increased at 1R-6 and 1R-24. During the second round of cold stress-recovery, T-AOC significantly increased at most time points except 2S-1 (Figure 3B). However, when compared to the prestressed status, T-AOC did not significantly change during the second cold stress and recovery, suggesting that the prestressed T-AOC should have been optimized or served as a new baseline to cope with the repeated environmental changes (Supplementary Table S1).

#### Response of Organismal Antioxidative Indicators

**FIGURE 4:**
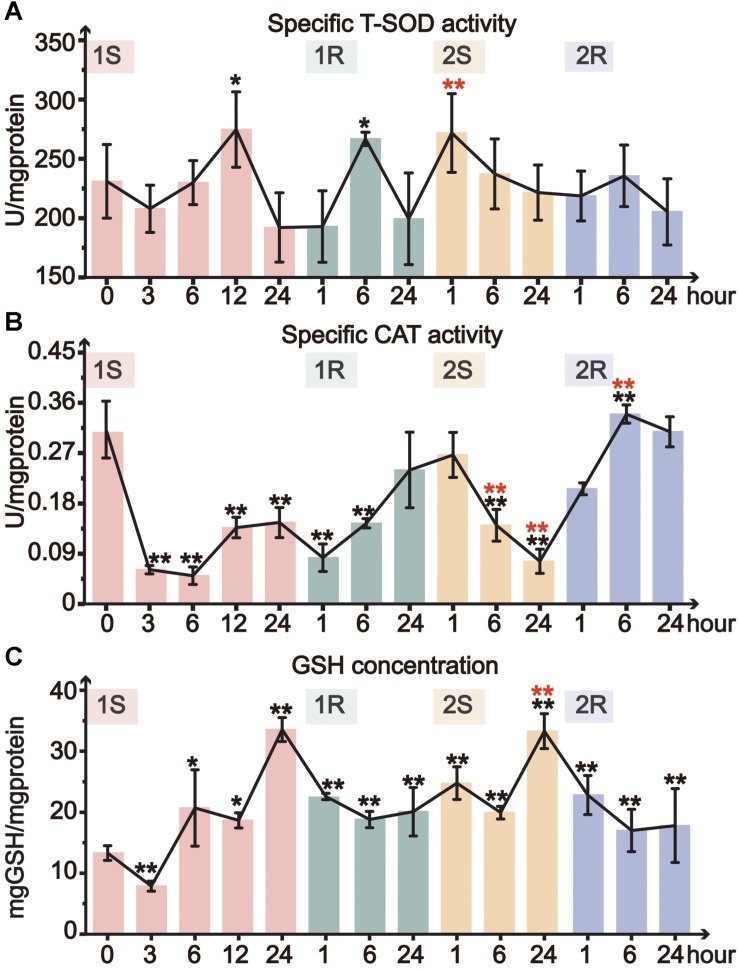
Organismal antioxidative indicators of *Ciona robusta* under unstressed (20°C), cold challenge (5°C) and recovery (20°C). **(A)** T-SOD (total superoxide dismutase) activity; **(B)** CAT (catalase) activity; **(C)** reduced GSH (glutathione) concentration; Each error bar represents the mean ± SE (*N* = 6). In the second round, the significant difference between the unstressed and prestressed was represented by black and red color, respectively. *p < 0.05, **p < 0.01.

Total superoxide dismutase activity only significantly increased at 1S-12 and 1R-6, and then remained at the unstressed level throughout all subsequent treatments (Figure 4A). CAT activity significantly decreased during cold exposures and got resilience at recovery stages. The minimum value of CAT activity in the two rounds dropped to 16 and 18% of the unstressed value at 1S-6 and 2S-24, respectively (Figure 4B). While during the recovery stages, CAT activity slightly recovered and returned to the unstressed level at the end of both rounds (Figure 4B). When comparing the response between two rounds of cold stress and recovery, we found that CAT activity slowly decreased in Round II with a delayed response, suggesting the increased tolerance in Round II.

The concentration of GSH significantly increased during cold stresses while decreased at recovery stages in both rounds (Figure 4C). The GSH content increased with the stress duration and showed the highest value at the end of each stress (1S-24 and 2S-24) with similar fold change (3-fold). Whereas, the decline of GSH content during recovery stages did not show complete resilience but was still significantly higher than the unstressed level. In Round II, GSH content was maintained at a significantly elevated prestressed level and showed significant increase in 2S-24 relative to the prestressed status (Supplementary Table S1). All these results indicate that *C. robusta* re-adjusted the status with the elevated GSH content to antioxidative for subsequent cold challenges.

Given that the experimental temperature was sublethal for *C. robusta* (Dybern, 1965; Marin et al., 1987), the results here confirmed that the used conditions were not chronic stress but mutually independent repeated stresses. Hence, the steady antioxidative levels under the repeated stresses revealed the important role of the physiological plasticity of ADS for homeostasis.

### Transcriptional Response of ADS-Related Genes

#### General Patterns of Transcriptional Changes

The gene expression data was analyzed using PCA to examine the general pattern of transcriptional changes. PCA results showed that three axes explained 87.7% of the total variation of transcriptional changes (Figure 2B). Similarly to the results on physiological changes, samples collected in cold stresses were separated from those in unstressed and recovery phases by PC2 (13.3%). Meanwhile, unstressed ascidians were clustered separately from those in recovery phases by PC3 (9.1%). Given the consideration between two stress phases, samples collected in the second stress were an overlap of those in the first stress with a more concentrated distribution. Such a pattern suggest that the response amplitude of the repeated stress should be narrower than that of the initial response and the recurrent response should be weak (i.e., cold stress memory).

#### Expression of Downstream Genes of Nrf2-keap1 Signal Pathway

**FIGURE 5:**
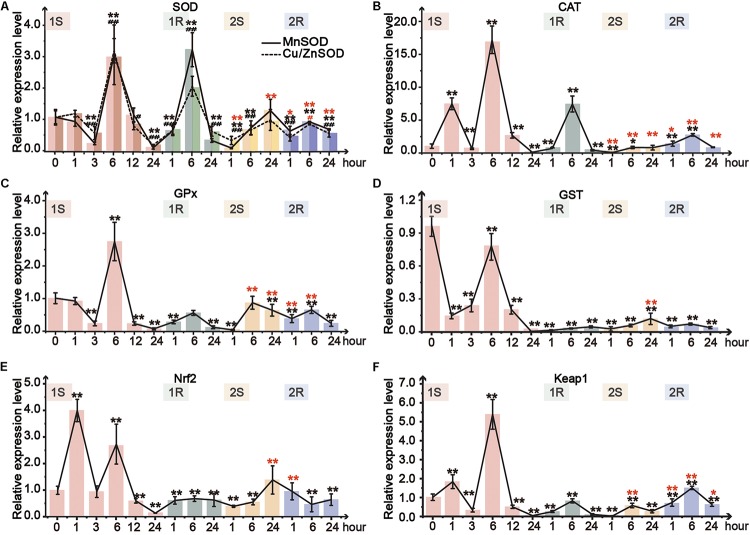
Relative expression level in pharynx muscle of *Ciona robusta* under unstressed (20°C), cold challenge (5°C) and recovery (20°C). **(A)** superoxide dismutase (SOD); **(B)** catalase (CAT); **(C)** glutathione S-transferases (GSTs); **(D)** glutathione peroxidase (GPX); **(E)** Nuclear factor (erythroid-derived 2)-like 2 (Nrf2); **(F)** Kelch-like ECH-associated protein 1 (Keap1). Each bar represents the mean ± SE (*N* = 6). In Figure 5A, significant difference of MnSOD and Cu/ZnSOD was represented by asterisk (*) and pound sign (**#**), respectively. In the second round, the significant difference between the unstressed and prestressed was represented by black and red color, respectively. */**^#^***p* < 0.05, **/**^##^***p* < 0.01.

**FIGURE 6:**
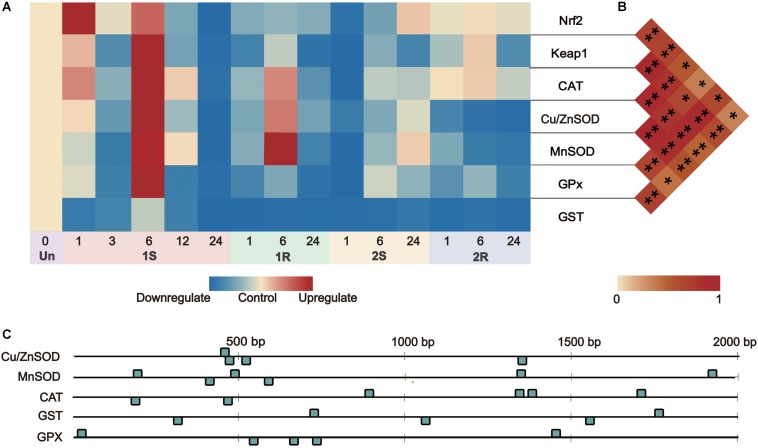
Gene expression and correlation between Nrf2 and its downstream genes under recurrent environment stress. **(A)** Heat map of gene expression, Un = unstressed; **(B)** Pair-wise Pearson correlation coefficients (*r*) between transcriptional factors Nrf2 and its downstream genes. **(C)** The numbers and distribution of identified ARE (antioxidant-responsive element) by core sequence 5′-G/ATGACNNNGC-3′ in downstream genes. *p < 0.05, **p < 0.01.

Amongst the TFs (Nrf2, Keap1) and downstream genes (MnSOD, Cu/ZnSOD, CAT, GST, and GPx), the expression range of Round II was narrower than that of Round I, suggesting the decreased response after prestressing and the appearance of cold stress memory (Figures 5, 6A).

Two isoforms of SOD, Cu/ZnSOD and MnSOD, showed similar response patterns (Figure 5A). The expression of both isoforms was tripled at 1S-6 and then sharply decreased at 1S-24. However, the two isoforms were merely upregulated to 1.1- and 1.2-fold in the second round of cold challenge, respectively. During recovery phases, the expression of these two SOD isoforms was significantly increased in the first 6 h (1R-1∼1R-6; 2R-1∼2R-6) and then declined at the end of each round. MnSOD was more sensitive and showed a robust response with a broader range than Cu/ZnSOD. Meanwhile, during the first round, CAT expression was significantly upregulated by 7. 5-, 17-, and 7.5-fold at 1S-1, 1S-6, and 1R-6, respectively, which was much higher than 1.5-fold at 2R-1 and 3-fold at 2R-6 (Figure 5B).

Two genes, GPx and GST, were selected to detect GSH metabolism. GPx expression showed 3-fold increase after 6-hour stress and then was significantly decreased to the value lower than that of the unstressed (Figure 5C). While in the second round, GPx was only increased to the unstressed level at 2S-6. The expression of GST was down-regulated during the whole experiment (Figure 5D). Meanwhile, the expression change range of the first round was between 0.1- and 0.8-fold, which were much wider than that of the second round (0.1- to 0.15-fold).

As the crucial molecular switch of Nrf2-keap1 signal pathway, the transcriptional expression of Nrf2 under cold exposure was sharply increased at 1S-1 and 1S-6 by 4- and 3-fold, respectively, and then significantly declined to the lowest value at 1S-24 (Figure 5E). During the first recovery stage, Nrf2 expression slowly recovered from the lowest value up to the similar value of the unstressed. The expression of Nrf2 was significantly decreased at 2S-1 and then continuously rose and stabilized to the prestress level. As the expression of Keap1 can be induced by the accumulation of Nrf2, similarly to Nrf2, the expression of Keap1 was only upregulated at 1S-1, 1S-6 and 2R-6 (Figure 5F).

In addition, given the comparison between the expression in the second round (six timepoints in total) and the unstressed, the expression of ADS-related genes at most timepoints (4/6 – 6/6) showed significant changes. When comparing the expression level of the second round to that of the prestressed, we found that expression of ADS-related genes showed significant changes at a fewer timepoints (1/6 - 5/6; Supplementary Table S2). For instance, expression of GST at all timepoints in the second round showed significant changes when compared to the unstressed value, while did not change when compared to the prestressed value except for 2S-24. Such a pattern demonstrated that the prestressing could remodel the baseline of gene expression, and the expression level during the second round was much closer to that after prestressing, but different from that of the unstressed.

#### Co-expression Relationship Between Nrf2 and Other Antioxidative Related Genes

By predicting the number of Antioxidant Response Element (ARE, DNA binding sites of Nrf2), we found that all the downstream genes on Nrf2-keap1 pathway had 4-6 ARE sites scattered along the 2000 bp upstream of each sequence (Figure 6C). Nrf2 expression was positively related to the expression level of Keap1, Cu/ZnSOD, CAT, GPx (*r* > 0.5, *p* < 0.05) and weakly related to MnSOD expression (*r* = 0.355, *p* = 0.032; Supplementary Table S3). This co-expression relationship between the transcription factor and its target genes indicated the potential “memory factor” role of Nrf2 to mediate the stress memory of its target genes in stress memory.

## Discussion

### Dynamic Response of the Antioxidant Defense System Under Cold Stress

Intense temperature changes such as sudden cold stress could largely disturb physiological conditions and metabolism of a species (Chen et al., 2019). Such adverse effects are highly related to the balance between generation and elimination of reactive oxygen species (ROS), which further triggers the activation of ADS (Sanchez-Nuno et al., 2018). Furthermore, cold-induced antioxidant defense largely relies on the dynamic changes of the activity and expression of ADS-related genes to cope with environmental changes (Wu et al., 2019). SOD is the first line in the elimination of ROS loads, since it can convert O^2–^ to O_2_ and H_2_O_2_, which is subsequently transformed into H_2_O by CAT and GPx (Ma, 2013). Our results showed the transient activation of T-SOD, which was consistent with the excessive accumulation of MDA at the same time points (Figures 3A, 4A). The results here suggest that the activation of T-SOD should be a protective mechanism to prevent excessive oxidation of polyunsaturated fatty acids (PUFAs) and defend against further damages (Hofmann and Todgham, 2010; Kammer et al., 2011). The stable status in most timepoints might be the result of redox balance between superoxide production and antioxidant defense (Chen et al., 2019). At the transcriptional level, our results showed that the response pattern of different SOD isoforms was consistent (Figure 5A), while the response amplitude of MnSOD was greater than that of Cu/ZnSOD. These results suggest that ROS loads should be eliminated by the synthesis of SOD to protect tissues from oxidative damages, and MnSOD was the major functional isoform. The different response amplitudes of two SOD isoforms might be related to their various cellular distributions (Pietsch, 2017). MnSOD converts H_2_O_2_ in the mitochondrial matrix space, which is the main place producing ROS and the most sensitive position suffering from ROS effects (Murphy, 2009; Sies et al., 2017). Although Cu/ZnSOD has been approved as the major isoform (95% of the total SOD) in cells, it functions in intermembrane space and might play a weak role in cleaning ROS (Shadel and Horvath, 2015; Wu et al., 2019).

The catalyzation of CAT and biotransformation of GSH have complementary roles in the decomposition of H_2_O_2_ (Lesser, 2006; Sthijns et al., 2016). GSH and H_2_O_2_ could be catalyzed to GSSH and H_2_O by GPx (Zhang et al., 2019). In our study, CAT activity showed significant decrease under cold stresses, while the GSH content and the expression of GPx gene were elevated (Figures 4C, 5B,C). These results suggest that *C. robusta* might largely rely on GSH and GPx to scavenge H_2_O_2_ and lipid hydroperoxides. Additionally, we observed discordant response patterns of CAT on enzyme activity and gene expression regulatory levels (Figures 4B, 5B), which might suggest the potential effects of post-transcriptional and post-translational regulation of CAT in response to recurrent cold challenges. Since the half-life period of CAT mRNA is rather long (about 42 h in human cell and 30 h in saltwater clam *Meretrix meretrix*), some unidentified redox sensitive proteins can bind to the 5′ UTR of the CAT mRNA to regulate its translational rate and plus at the protein level, as observed in rat PC12 cancer cells exposed to H_2_O_2_ (Haque et al., 2012). It has been also reported that human ARPE-19 retinal pigment epithelial cells under several stressors including H_2_O_2_ can alter miRNA expression profiles for ROS, of which miR-30b can bind to the 3′ UTR of CAT mRNA, leading to a drastic decrease of CAT protein (Glorieux et al., 2015; Leisegang et al., 2018). Post-translational modifications, such as phosphorylation and ubiquitinylation, can regulate the turnover and degradation of CAT enzyme (Cao et al., 2003). In human cancer cells exposed to oxidative stress (H_2_O_2_), tyrosine kinases c-abl (abelson murine leukemia viral oncogene homolog 1) and c-abl-related gene (Arg) were able to phosphorylate human CAT enzyme at residues Tyr231 and Tyr386 to inhibit the CAT at the enzyme activity level. Therefore, although CAT gene expression was upregulated under cold stresses, the enzyme activity of CAT was still decreased possibly due to its translational repression induced by epigenetic changes or later biochemical processes.

The induction of ADS-related genes is regulated by several signaling pathways, in which Nrf2-Keap1 is a master regulatory pathway (Ma, 2013). In the present study, the elevation of Nrf2 expression showed an active response, which was further confirmed by the significant positive correlation between the expression of Nrf2 and its downstream genes (Figure 6B). Nevertheless, the continuous expression of Nrf2 could cause negative effects, such as free radical damages and cell apoptosis (Zheng et al., 2016). The elevation of Keap1 expression could activate the feedback regulation and increase Nrf2 degradation to control Nrf2 abundance (Zhang, 2006). Our results showed coordinated increase in the expression of Nrf2 and Keap1 (Figures 5E,F), supporting the role of keap1 in facilitating the termination of the pathway timely and preventing the overexpression of genes in Nrf2-keap1 signaling pathway. The positive response and feedback regulation of genes on Nrf2-keap1 signaling pathway ensured the ADS to maintain the balance of reactive oxygen free radicals through a series of changes and promote adaptive homeostasis (Kensler et al., 2007). In addition, Nrf2 could orchestrate the regulation of the gene expression involved in innate immunity, which confers protection by modulation of inflammatory defensive response against various environmental stresses (Battino et al., 2018). Cold stress can induce the upregulation of Nrf2 transcripts, which elicit antioxidant response to keep redox balance. Meanwhile, as the regulator of the innate immune response, Nrf2 can regulate the expression of the transcription factor in NF-κB signaling pathway to inhibit the transcriptional activity of NF-κB and protect animals against the adverse environment (Thimmulappa et al., 2006). By simultaneously modulating ADS and innate immune system, Nrf2 could enhance the rapid response for the stress protection and furthermore potentially invoke inflammatory defense to tissue integrity/repair under fluctuating environments.

Our results showed that after the transient increase at the expression level (1S-6 and 2S-6), most cold-induced gene expressions returned to renewed status (i.e., the rebuilt baselines) after a short period of time (i.e., transcriptional resilience; Figure 5). Transcriptional resilience was an indicator to predict stress tolerance (Franssen et al., 2011). For example, the study on the resilient patterns of recurrent bleaching on corals (genus *Acropora*) showed that the resilience capacity after bleaching strongly contributed to the sensitivity to future bleaching, and the species *A. gemmifera* with a fast recovery speed resulted in a high level of survival under repeated heat extremes (Thomas et al., 2019). Similarly, the transcriptional resilience in this study adds evidence on the adaptative response against recurrent stresses and should be functional in adaptative homeostasis simultaneously.

### Cold Stress Memory Induced by Recurrent Stresses

Both the decreased response amplitude of ADS-related gene expression (Figure 5) and enhanced antioxidant capacity (Figure 3B) illustrated that *C. robusta* obtained cold stress memory to maintain adaptive homeostasis. Stress memory has been detected in multiple species and was characterized by decreased reactivity (tolerance) or enhanced response (potentiation) to subsequent challenges (Avramova, 2015). Study on murine embryonic fibroblasts (MEF) showed that pre-exposure to H_2_O_2_ could increase cell counts, BrdU incorporation and protein carbonyl content in subsequent challenges (Pickering et al., 2013). However, the transcriptional stress memory of *A. thaliana* was characterized by the expression decrease of osmotic and ionic equilibrium related genes under recurrent drought stresses (Ding et al., 2013). From a broad perspective, stress memory is a mechanism for organisms to store the message from an initial stress and becomes more robustness in response to future stresses, leading to the tolerating, priming, or acclimation affects after prestressing (Bruce et al., 2007).

Studies have proposed several molecular mechanisms underpinning stress memory. One mechanism was proposed to be sustained modulations of key signaling metabolites or transcription factors, which could initiate cascaded effects on downstream genes (Mozgova et al., 2019). For instance, recurring heat stresses induced the sustained expression of miR156, which regulated the transcriptional factor, Squamosa Promoter Binding Protein-Like (SPL), in *A. thaliana*, affecting endogenous timing of leaf initiation and developmental transitions (Stief et al., 2014). Another possible avenue was proposed to rely on chromatin modifications and epigenetic marks. Flowering Locus C (FLC) was transcriptionally repressed by epigenetic silence of H3K27me3 under cold exposure, and repression was then epigenetically consolidated during subsequent warmer temperature, facilitating a memory of cold challenges (Berry and Dean, 2015).

Nuclear factor erythroid-2-related factor 2 signaling pathway involves the maintenance of redox balance and stress response regulation, and the upregulated Nrf2 could result in transcriptional activation of its downstream genes to protect against various environmental stresses (Ma, 2013). The significant positive correlation between the expression of Nrf2 and ADS-related genes (Figure 6B; Supplementary Table S3), as well as the presence of ARE in the promoter regions of ADS-related genes (Figure 6C), illustrated the initiation of the Nrf2 pathway and implied the potential regulatory relationship between Nrf2 and other ADS-related genes. Considering sustained/accumulated expression levels of transcription factors for stress memory, the sustained changes of Nrf2 expression would potentially be related to the cold stress memory of ADS. For the potential mechanism of cold stress memory in this study, Nrf2 could act as the key transcription factor and potential “memory factor” whose sustained expression could regulate the transcriptional level of ADS-related genes during the subsequent cold exposure. The evidence obtained in this study provides baselines for further investigation of transcription factor patterns to reveal dynamic changes of ADS for comprehensive understanding of stress memory under environmental stresses.

### The Effects of Cold Stress Memory

The acquisition of stress memory has several benefits, such as effectively resisting recurring environmental stresses and remaining homeostasis status (Ding et al., 2013; Avramova, 2015; Virlouvet and Fromm, 2015; Mozgova et al., 2019). Studies have approved that cells after prestressing were considerably more tolerant than non-prestressed cells to defend oxidative stresses in later challenges (Wiegant et al., 2011). Coral species experiencing heat stresses in previous years could reduce mortality during later heat shocks (Hughes et al., 2018; Harrison et al., 2019). Because organisms could modulate their internal states, such as energy allocation to activate/inactivate the expression of related genes and to coordinate physiological needs (López-Maury et al., 2008), the stress memory could shrink the energy allocation of ADS-related genes in recurrent stresses. Such a process allows prestressed individuals to cost less than those unstressed in response to future stresses. In the context of trade-off on resource allocation, stress memory could facilitate the expression of genes related to other functions such as growth and reproduction (van Hulten et al., 2006). Thus, stress memory provides an energy-efficient strategy to balance gene functions, which would possibly promote the homeostasis from rapid response to evolutionary adaptation (López-Maury et al., 2008).

Stress memory might promote the adaptive capacity to cope with harsh environments, making positive contributions to enlarging the ecological amplitude of a species. Similar to the function of feedback regulation in Nrf2-Keap1 signaling pathway, stress memory could limit the adverse impacts on overexpressed or persistent activation in the function of a protective effect against harsh environments (Melillo et al., 2018). Persistent adaptive response can be triggered by the duration of stresses, which can further increase the tolerance and make the physiological changes reduced gradually (potentially cause desensitization; Muniz-Castillo et al., 2019).

However, stress memory could lead to continuous resource consumption to maintain the readjusted baseline (i.e., stress memory can also entail costs; van Hulten et al., 2006). It is obvious that in an ambient condition (i.e., no disturbance), the ADS is always under dynamic balance and an organism has the highest fitness (Ristow, 2014). We assume that after prestressing, the redox balance would be no more disrupted until recurrent stresses appear, and the fitness of prestressed individuals would not be higher than that of unstressed ones. Unless the repeated challenges occur, organisms would undertake the costs for maintaining a remodeled baseline and merely gain growth benefits. Therefore, the recurring environmental pressures would be preconditions for an organism to get the opportunity to gain benefits from stress memory. Thus, when judging whether stress memory is the optimal strategy for survival of a species, we should consider the frequency of stress occurrence, as well as the associated benefits and costs (Maleck et al., 2000; van Hulten et al., 2006).

### Potential Roles of Cold Stress Memory in Invasions

Our recurrent cold stress/recovery treatments on *C. robusta* simulated the fluctuating environments during the process of biological invasions. Usually, great and/or fast environmental challenge is an inescapable threat for invasive species. Stress memory might exert fundamental influence in response to future challenges, as the enduring effects of prior experience along with changing environments unfold over time (Hughes et al., 2018). At the early stages of invasions, *C. robusta* could both complete the geographical shifts and remain adaptive homeostasis by successfully invoking ADS to defense stresses. Additionally, organisms could effectively store “memory (rebuilding the baseline of ADS status)” from experienced stresses and optimize stress response at subsequent invasion stages. Stress memory could promote response by shrinking the amplitude of transcriptional changes, as well as by allocating limited resources to ADS related genes. In this perspective, stress memory can strongly impel to the process of later population establishment, as it probably tunes the resource allocation in favor of other functions such as growth and reproduction. We conclude that stress memory is a strategy to stabilize the status of ADS after multiple stresses, and it is imperative to lucubrate possible mechanisms of stress memory to deeply understand rapid adaptation during biological invasions.

## Conclusion

Our results showed *C. robusta* relied on the resilience of ADS to maintain the adaptive homeostasis against environmental challenges. The resilience process of ADS transiently elevated the enzyme activities and transcription of ADS-related genes to readjusted baselines. The readjustment of ADS status after the initial stress was regarded as an active response to result in the stress memory. Subsequently, stress memory largely decreased the physiological response to recurrent environmental challenges. Overall, stress memory was an optimized strategy to allow *C. robusta* to store the “information” derived from initial invasion stages and decreased the subsequent responses to promote the homeostasis in the invasion process. Our results provide new insights into the mechanism of rapid physiological adaption under recurrent stresses and maintenance of adaptive homeostasis during biological invasions.

## Data Availability Statement

All datasets generated for this study are included in the article/Supplementary Material.

## Author Contributions

All authors conceived the study, designed the experiments, and took part in the interpretation of results and preparation of the manuscript. HL performed the experiments.

## Conflict of Interest

The authors declare that the research was conducted in the absence of any commercial or financial relationships that could be construed as a potential conflict of interest.

## Footnotes

1 http://asia.ensembl.org/index.html

2 http://www.premierbiosoft.com/primerdesign/index.html

3 http://www.genomatix.de
