# Supplementary material for: Stress Memory of Recurrent Environmental Challenges in Marine Invasive Species: Ciona robusta as a Case Study
Source: Front Physiol. 2020 Feb 13;11:94. doi: 10.3389/fphys.2020.00094 (PMC7031352; doi:10.3389/fphys.2020.00094)
Supplement: Supplementary file 1 [file Table_1.DOCX]

**Supplementary Table 1.** The difference of enzyme activity during the second round of cold stress using one-way ANOVA. Un = unstressed, Pre = prestressed; *: *p* < 0.05, **: *p* < 0.01.

|  | **MDA** | | **T-AOC** | | **T-SOD** | | **CAT** | | **GSH** | |
| --- | --- | --- | --- | --- | --- | --- | --- | --- | --- | --- |
|  | **Un** | **Pre** | **Un** | **Pre** | **Un** | **Pre** | **Un** | **Pre** | **Un** | **Pre** |
| 1S-3 | 0.07 |  | 0.39 |  | 0.31 |  | 0.00** |  | 0.01** |  |
| 1S-6 | 0.89 |  | 0.52 |  | 0.96 |  | 0.01** |  | 0.04* |  |
| 1S-12 | 0.01* |  | 0.01** |  | 0.04* |  | 0.01** |  | 0.05* |  |
| 1S-24 | 0.87 |  | 0.98 |  | 0.09 |  | 0.01** |  | 0.00** |  |
| 1R-1 | 0.88 |  | 1.00 |  | 0.12 |  | 0.00** |  | 0.01** |  |
| 1R-6 | 0.04* |  | 0.01** |  | 0.05* |  | 0.01** |  | 0.01** |  |
| 1R-24 (pre) | 0.62 |  | 0.01** |  | 0.20 |  | 0.35 |  | 0.01** |  |
| 2S-1 | 0.32 | 0.64 | 0.27 | 0.15 | 0.10 | 0.01** | 0.96 | 0.41 | 0.01** | 0.24 |
| 2S-6 | 0.87 | 0.54 | 0.01** | 0.22 | 0.79 | 0.15 | 0.00** | 0.01** | 0.00** | 0.89 |
| 2S-24 | 0.35 | 0.07 | 0.01** | 0.31 | 0.70 | 0.40 | 0.00** | 0.00** | 0.00** | 0.00** |
| 2R-1 | 0.64 | 0.98 | 0.01* | 0.42 | 0.61 | 0.46 | 0.08 | 0.42 | 0.00** | 0.99 |
| 2R-6 | 0.72 | 0.90 | 0.01** | 0.35 | 0.85 | 0.17 | 0.01** | 0.00** | 0.00** | 0.15 |
| 2R-24 | 0.70 | 0.33 | 0.01** | 0.25 | 0.30 | 0.82 | 0.30 | 0.00** | 0.00** | 0.26 |

**Supplementary Table 2.** The difference of gene expression during the second round of cold stress using one-way ANOVA. Un = unstressed, Pre = prestressed; *: *p* < 0.05, **: *p* < 0.01.

|  | **MnSOD** | | **Cu/ZnSOD** | | **CAT** | | **GPX** | | **GST** | | **Nrf2** | | **Keap1** | |
| --- | --- | --- | --- | --- | --- | --- | --- | --- | --- | --- | --- | --- | --- | --- |
|  | **Un** | **Pre** | **Un** | **Pre** | **Un** | **Pre** | **Un** | **Pre** | **Un** | **Pre** | **Un** | **Pre** | **Un** | **Pre** |
| 1S-1 | 0.28 |  | 0.17 |  | 0.00** |  | 0.70 |  | 0.00** |  | 0.00** |  | 0.00** |  |
| 1S-3 | 0.00** |  | 0.00** |  | 0.00** |  | 0.01** |  | 0.00** |  | 0.56 |  | 0.00** |  |
| 1S-6 | 0.00** |  | 0.00** |  | 0.00** |  | 0.00** |  | 0.00** |  | 0.00** |  | 0.00** |  |
| 1S-12 | 0.12 |  | 0.03* |  | 0.01** |  | 0.01** |  | 0.01** |  | 0.01** |  | 0.01** |  |
| 1S-24 | 0.00** |  | 0.00** |  | 0.00** |  | 0.00** |  | 0.00** |  | 0.00** |  | 0.00** |  |
| 1R-1 | 0.00** |  | 0.00** |  | 0.00** |  | 0.00** |  | 0.00** |  | 0.00** |  | 0.00** |  |
| 1R-6 | 0.00** |  | 0.00** |  | 0.00** |  | 0.00** |  | 0.00** |  | 0.00** |  | 0.00** |  |
| 1R-24 (pre) | 0.00** |  | 0.00** |  | 0.00** |  | 0.00** |  | 0.00** |  | 0.00** |  | 0.00** |  |
| 2S-1 | 0.00** | 0.00** | 0.01** | 0.34 | 0.00** | 0.00** | 0.00** | 0.33 | 0.00** | 0.45 | 0.00** | 0.15 | 0.00** | 0.42 |
| 2S-6 | 0.00** | 0.63 | 0.05* | 0.24 | 0.02* | 0.01** | 0.10 | 0.00** | 0.00** | 0.61 | 0.00** | 0.65 | 0.00** | 0.00** |
| 2S-24 | 0.31 | 0.00** | 0.59 | 0.27 | 0.21 | 0.00** | 0.00** | 0.00** | 0.00** | 0.00** | 0.23 | 0.00** | 0.00** | 0.13 |
| 2R-1 | 0.00** | 0.03* | 0.02* | 0.11 | 0.00** | 0.05* | 0.00** | 0.00** | 0.00** | 0.86 | 0.89 | 0.00** | 0.01* | 0.00** |
| 2R-6 | 0.00** | 0.00** | 0.57 | 0.05* | 0.00** | 0.00** | 0.00** | 0.00** | 0.00** | 0.30 | 0.00** | 0.32 | 0.00** | 0.00** |
| 2R-24 | 0.00** | 0.00** | 0.01* | 0.78 | 0.23 | 0.00** | 0.00** | 0.15 | 0.00** | 0.79 | 0.00** | 0.89 | 0.00** | 0.04* |

**Supplementary Table 3.** The correlation coefficients (*r,* above diagonal) and *P* values (*p,* below diagonal) of the Co-expression analysis. *: *p* < 0.05, **: *p* < 0.01

| *r p* | Nrf2 | Keap1 | CAT | MnSOD | Cu/ZnSOD | GST | GPX |
| --- | --- | --- | --- | --- | --- | --- | --- |
| Nrf2 |  | 0.676** | 0.679** | 0.531* | 0.355* | 0.649* | 0.541* |
| Keap1 | 0.008 |  | 0.942** | 0.817** | 0.635* | 0.962** | 0.895** |
| CAT | 0.008 | 4.68E-07 |  | 0.929** | 0.814** | 0.904** | 0.830** |
| Cu/ZnSOD | 0.049 | 3.66E-04 | 1.52E-06 |  | 0.933** | 0.846** | 0.782** |
| MnSOD | 0.032 | 0.015 | 3.99E-04 | 1.13E-06 |  | 0.681** | 0.572* |
| GPx | 0.012 | 4.27E-08 | 9.27E-06 | 1.39E-04 | 0.007 |  | 0.875** |
| GST | 0.045 | 1.51E-05 | 2.41E-04 | 9.42E-04 | 0.032 | 4.12E-05 |  |
